# IFN-α-mediated Base Excision Repair Pathway Correlates with Antiviral Response Against Hepatitis B Virus Infection

**DOI:** 10.1038/s41598-017-13082-z

**Published:** 2017-10-05

**Authors:** Yong Li, Yuchen Xia, Meifang Han, Guang Chen, Dake Zhang, Wolfgang E. Thasler, Ulrike Protzer, Qin Ning

**Affiliations:** 1 Department and Institute of Infectious Diseases, Tongji Hospital, Tongji Medical College, Huazhong University of Science and Technology, Wuhan, 430030 China; 2 Institute of Virology, Technical University of Munich / Helmholtz Zentrum München, 81675 Munich, Germany; 3 Liver Diseases Branch, National Institute of Diabetes and Digestive and Kidney Diseases (NIDDK), NIH, 20892 Bethesda, Maryland USA; 4 Key Laboratory of Genomic and Precision Medicine, Beijing Institute of Genomics, Chinese Academy of Sciences, Beijing, 100101 China; 5 Department of General, Visceral, Transplantation, Vascular and Thoracic Surgery, Grosshadern Hospital, Ludwig Maximilians University, 81377 Munich, Germany; 6 German Center for Infection research (DZIF), Munich, Germany

## Abstract

Previous studies identified APOBEC deaminases as enzymes targeting hepatitis B virus (HBV) DNA in the nucleus thus affecting its persistence. Interferon (IFN)-α treated chimpanzees and hepatitis C patients showed elevated APOBEC expression. We thus hypothesized that the responses to IFN-α treatment of chronic hepatitis B (CHB) patients is influenced by IFN-induced base excision repair (BER). CHB-treatment naïve patients, patients treated with PEGylated IFN-α, and patients with sequential treatment of Entecavior and PEGylated IFN-α were recruited. Blood and liver biopsy samples were collected before treatment and at treatment endpoint. BER genes were assessed by quantitative RT-PCR. BER gene expression levels and IFN treatment responses were correlated in patient liver biopsies. APOBEC3A, -B, -C, -D/E, and-G mRNA levels were up-regulated in IFN-treated patients. APOBEC3A expression was significantly higher in IFN-responders than in non-responders. BER genes NEIL3 was down-regulated in IFN-treated patients. APOBEC3 and BER gene expression at treatment endpoints partially correlated with the corresponding absolute DNA level or degree of HBsAg and HBV DNA decline. Our study suggests that the expression of APOBEC3A positively correlates with IFN-treatment responses in CHB patients, while NEIL3 shows negative correlation. These genes may involve to IFN mediated viral suppression and serve as biomarkers for CHB disease management.

## Introduction

Hepatitis B remains a global health problem^1^, with an estimated 250–400 million people chronically infected with HBV worldwide^2^ at risk to develop end-stage liver disease such as HBV-induced liver failure, liver cirrhosis or hepatocellular carcinoma (HCC)^3,4^. Currently, there are two major classes of antivirals approved for the treatment of CHB: oral nucleos(t)ide analogues and immunomodulatory agents including unmodified and PEGylated interferon-α (PegIFN-α). These treatments can control virus replication, minimize liver damage and reduce the risk of HBV associated liver disease. Unfortunately, none of the available treatments can efficiently cure hepatitis B, because of the persistence of HBV covalently closed circular DNA (cccDNA) in the nucleus of infected cells^5^.

Nucleos(t)ide analogues inhibit HBV reverse transcription taking place before HBV egress and have no direct effect on the nuclear viral transcriptional template, the cccDNA. IFN-α has been a backbone of therapy of chronic hepatitis B (CHB)^6^. Dosing, however, is limited by side effects and response rates are less than 30% in CHB patients^4^. Although the molecular mechanism remained elusive, it remains the only drug leading to viral clearance^7^. In different experimental models, IFN-α induced products have been shown to restrict HBV infection at multiple steps^8,9^. Recently, it has been shown that in HBV infection cell culture models, induction of APOBEC3A and the base excision repair (BER) pathway by IFN-α or T-cell cytokines is essential for cccDNA decreasing^8,10,11^. IFN-α induced APOBEC3A deaminates the nuclear HBV DNA minus strand, resulting uracil-containing DNA that recognized, cleaved and degraded by cellular BER enzymes. Although APOBEC3A was found to be up-regulated in IFN-α treated chimpanzee and chronic hepatitis C patient livers^10^, the expression profiles of APOBEC3 family members as well as BER genes and their correlation with IFN-α treatment response in patients with CHB has not been addressed. In this study, we report that in particular the expression levels of DNA-editing enzymes APOBEC3A, but also BER gene NEIL3 correlate with response to IFN treatment in CHB patients. These genes indicate the molecular pathways involved in HBV cure but may also serve as biomarkers for CHB disease management.

## Results

### IFN-α treatment induces APOBEC3 mRNA expression in liver samples from CHB patients

**Figure 1:**
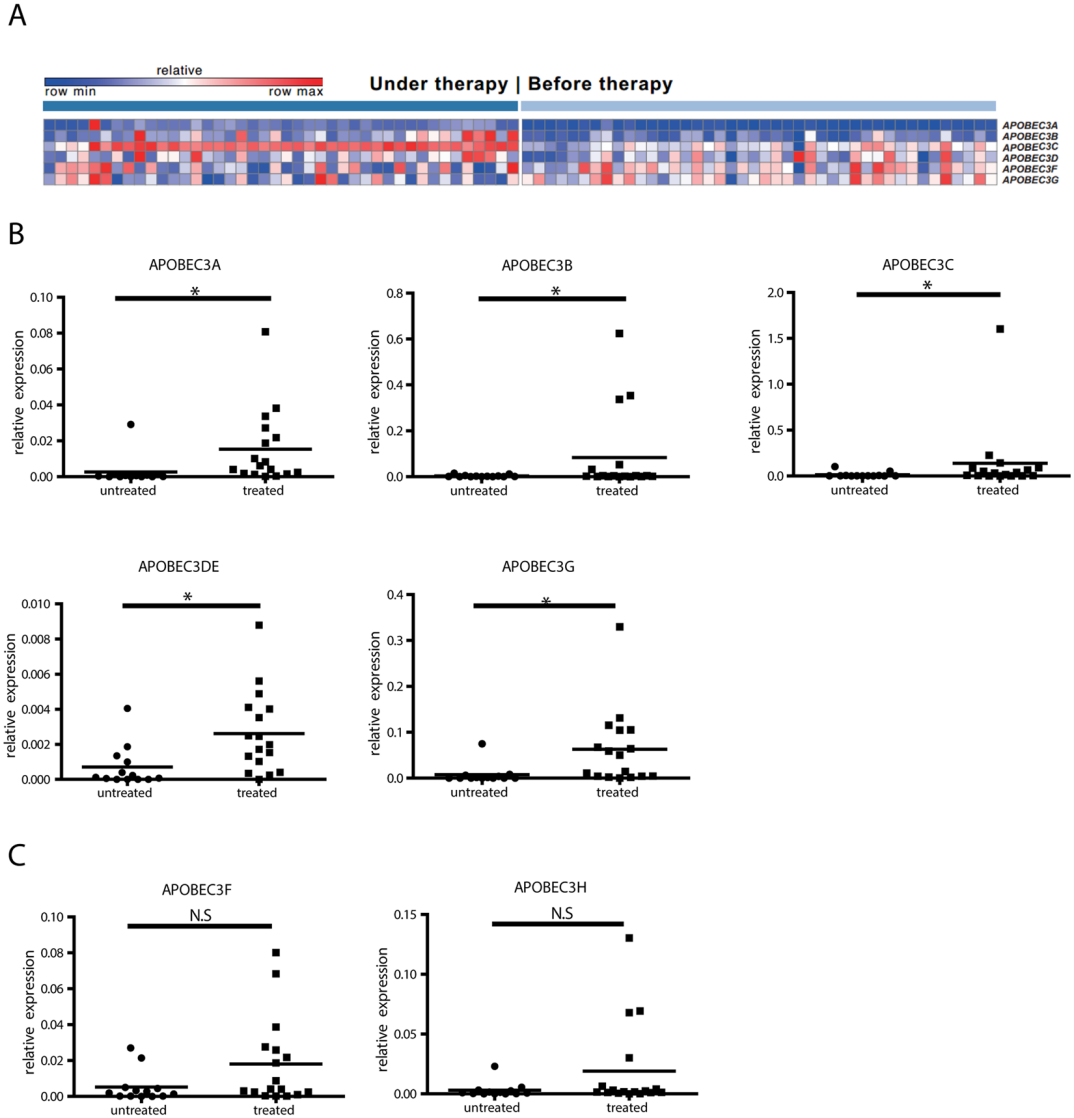
mRNA expression of APOBEC3 family members in liver biopsies of patients treated with IFN. (**A**) Meta analysis of transcription levels of APOBEC3 family members, in two studies reporting hepatitis C patients under IFN treatment^13,14^. The top scaled color bar indicates the relative gene expression level, and the dark and light blue bars illustrate two groups of patients: under and before therapy. In the heatmap, each column contains the data from one patient, and each square shows the gene expression level in a specific individual. All changes had FDR(BH) <0.01 and *p* after Bonferroni correction <0.05. More details for all six genes are summarized in Supplementary Table 3. (**B**,**C**) Total RNA from liver biopsies of naïve- or IFN-α-treated CHB patients were extracted. After reverse transcription, mRNA expression levels of APOBEC3A, -B, -C, -D/E, -G (**B**), -F, and -H (**C**) were quantified and normalized to GAPDH mRNA. Student’s *t* test was used to compare the two groups. *P < 0.05; N.S. no significance.

It is well acknowledged that IFN-α induces APOBEC3 family cytidine deaminases in cell culture models^10,12^, but whether IFN-α can induce APOBEC3 gene in liver biopsies from patients has not been conclusively studied. Due to the lack of available CHB patient data, we first did a meta analysis of two published datasets from hepatitis C patients^13,14^. In these two studies, liver biopsies of different individuals before and under interferon therapy were analyzed using microarray based expression profiling. As shown in Fig. 1A, among the APOBEC3 family members, APOBEC3A, APOBEC3B and APOBEC3C demonstrate increased expression levels in under therapy samples with fold changes at 14, 77.4 and 2.8 respectively. (p after Bonferroni correction <0.05) when comparing their expression before and under IFN therapy.

To determine whether similar expression patterns can be observed in CHB patients under IFN treatment, liver biopsies from CHB patients with or without IFN treatment were collected and qRT-PCR was performed (Supplementary Table 1). Hereby, we found that APOBEC3A, -B,-C,-DE, and -G mRNA expression were significantly induced in livers of patients treated with IFN-α compared to treatment-naïve patients or OSST patients^1^ before IFN-α treatment (Fig. 1B), while APOBEC3F and APOBEC3H were only up-regulated in three patients (Fig. 1C). We also analyzed paired samples only as shown in Fig. S1, APOBEC3A, -B and -G mRNA expression was significantly induced after IFN-α treatment. These findings indicate that APOBEC3 deaminases are regulated in livers of CHB patients following IFN-α treatment.

### Expression of APOBEC3A mRNA correlates with response to interferon treatment

**Figure 2:**
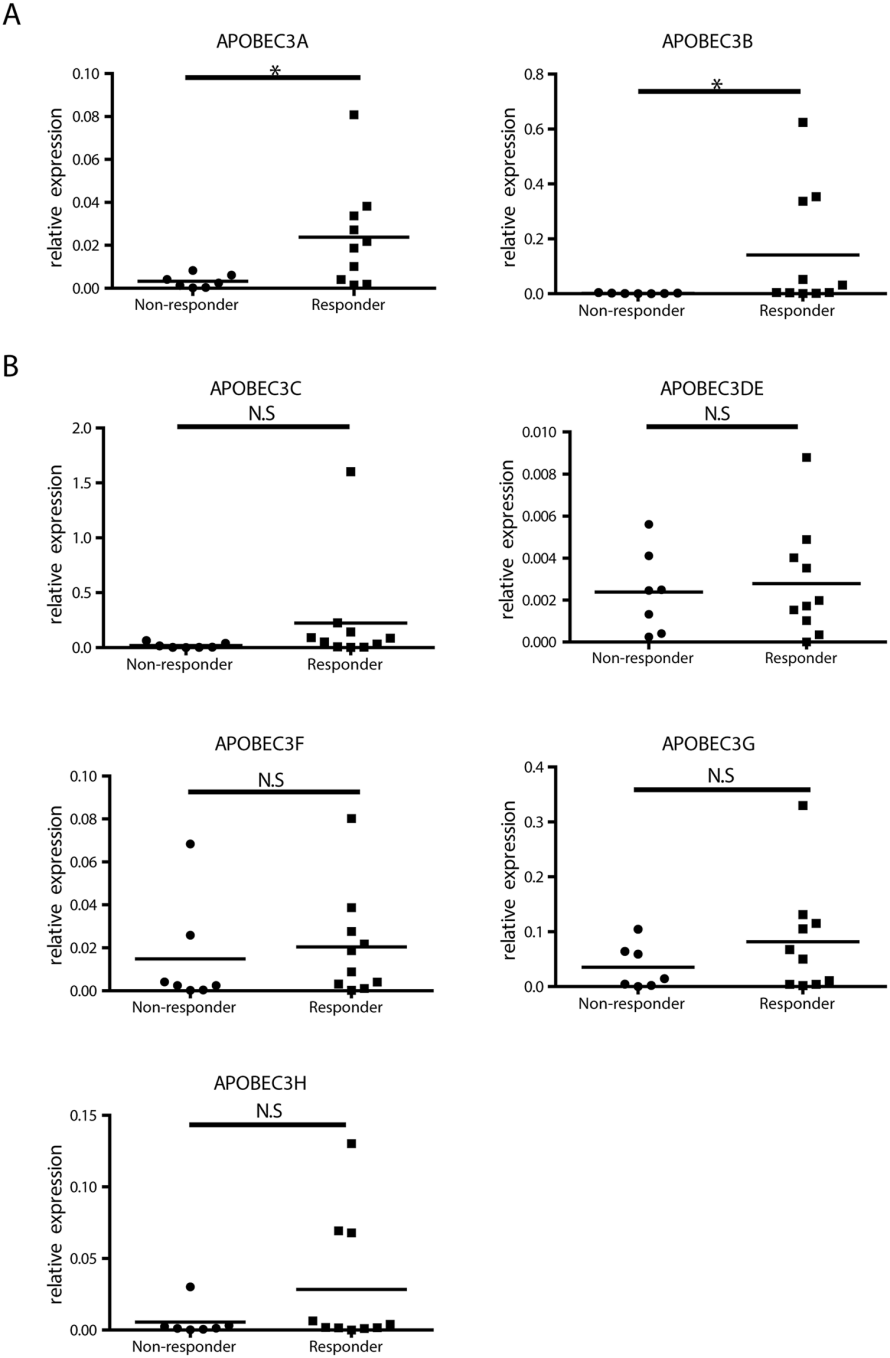
Comparison of APOBEC3 gene expression levels between responders and non-responders. Relative expression level of hepatic APOBEC3A (**A**), or APOBEC3B, -C, -DE, -F, -G, -H mRNA (**B**) normalized to GAPDH were compared in responders and non-responders at the end of PegIFN-α treatment. Student’s *t* test or Mann Whitney U test was used to compare the two groups. *P < 0.05; N.S., no significance.

The next question we wanted to answer is whether the expression of APOBEC3 deaminases correlate with the outcome of IFN-α therapy. To answer that question, we separated and compared the gene expression level of responders and non-responders to IFN-α treatment (response to therapy is defined as HBV DNA level <1000 copies/ml with HBeAg or HBsAg seroconversion or HBsAg decline >1Log_10_IU/ml at treatment endpoint). Interestingly, APOBEC3A and APOBEC3B mRNA levels were significantly higher in responders than in non-responders (Fig. 2A). While there was a tendency for increased APOBEC3C, APOBEC3G and APOBEC3H expression, expression of the other APOBEC3 genes did not differ between responders and non-responders (Fig. 2B). This indicated a role of *APOBEC3* deaminases, particularly APOBEC3A and APOBEC3B, for the outcome of interferon treatment.

**Figure 3:**
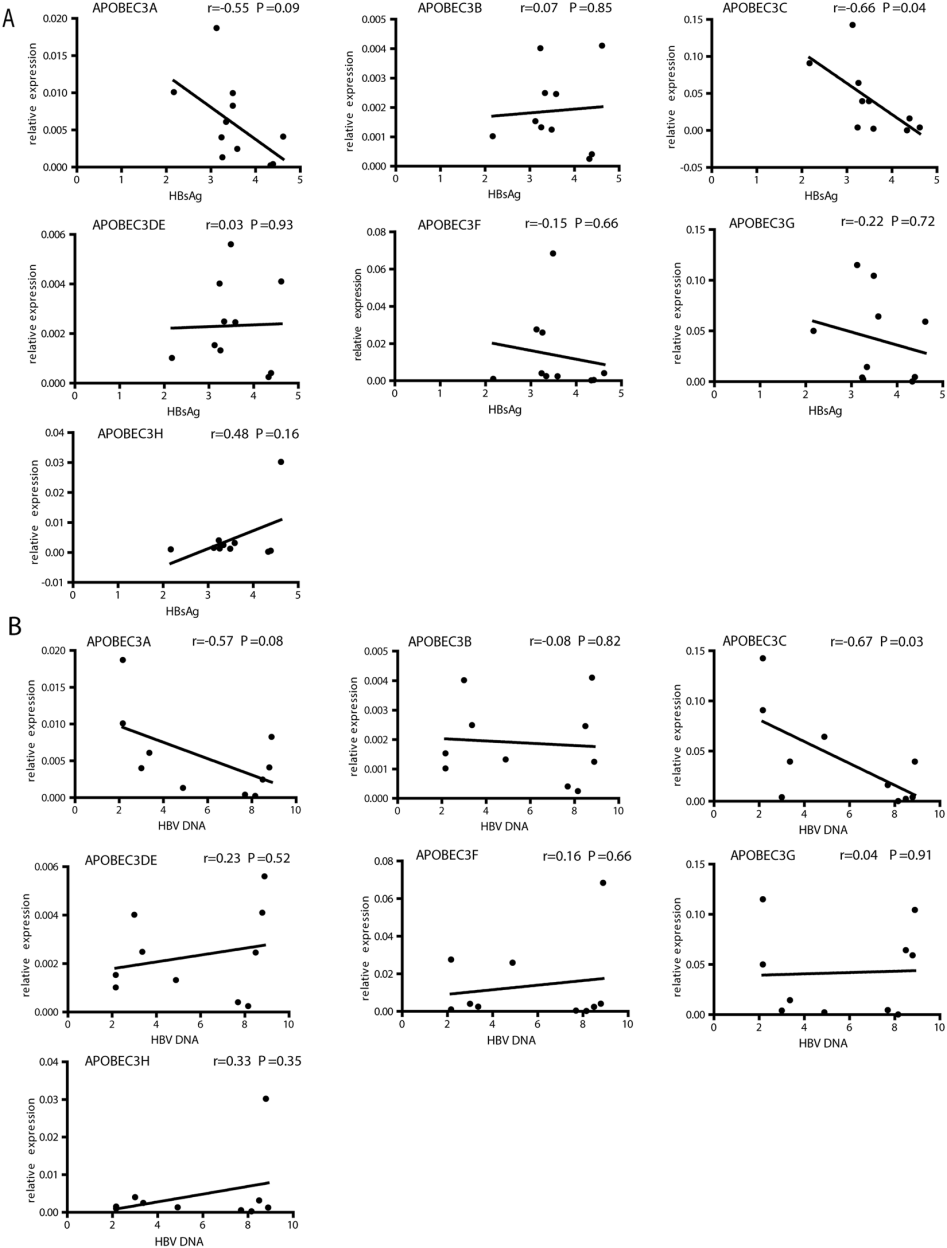
Correlation between *APOBEC3* gene expression and HBV markers. Correlations of hepatic APOBEC3 gene expression at treatment endpoint with HBsAg (**A**) or HBV DNA (**B**) in the patient serum at treatment endpoint were evaluated using Pearson correlation. Log 10 values of HBsAg or HBV DNA were bloted.

To further investigate whether APOBEC3 gene expression correlated with viral suppression, correlations of their expression and HBV biomarkers were studied. Since OSST patients’ HBV DNA were all below detection level before IFN therapy, antiviral response of only patients with IFN monotherapy were evaluated. Correlations between APOBEC3 expression levels and HBsAg or HBV DNA decline (Log _week 0_ − Log _week 48/96_) (Fig. S2). Hereby, APOBEC3F mRNA levels at treatment endpoint significantly correlated with the degree of HBsAg decline (Fig. S2A) and that of APOBEC3C with HBV DNA decline (Fig. S2B **)**, while APOBEC3A, -C, and -G showed a trend for correlation. At treatment endpoints, APOBEC3 gene mRNA level partially correlated with corresponding HBsAg or HBV DNA levels (Fig. 3, given in a Log10 scale). While APOBEC3A and -C mRNA level at treatment endpoints negatively correlated with HBsAg levels (p = 0.09 and p = 0.04, respectively), other APOBEC3 genes show modest or not correlation (Fig. 3A). Similar correlations were found between APOBEC3 mRNA levels and viremia at end of treatment (Fig. 3B). Higher HBV DNA associated with lower hepatic APOBEC3A and –C expression levels (p = 0.08 and p = 0.03, respectively).

Together, these data from clinical setting of patients suggest that higher expression of in particular APOBEC3A and -C indicate a better response to IFN-α during CHB treatment.

### IFN-α treatment regulates BER enzymes in liver samples from CHB patients

**Figure 4:**
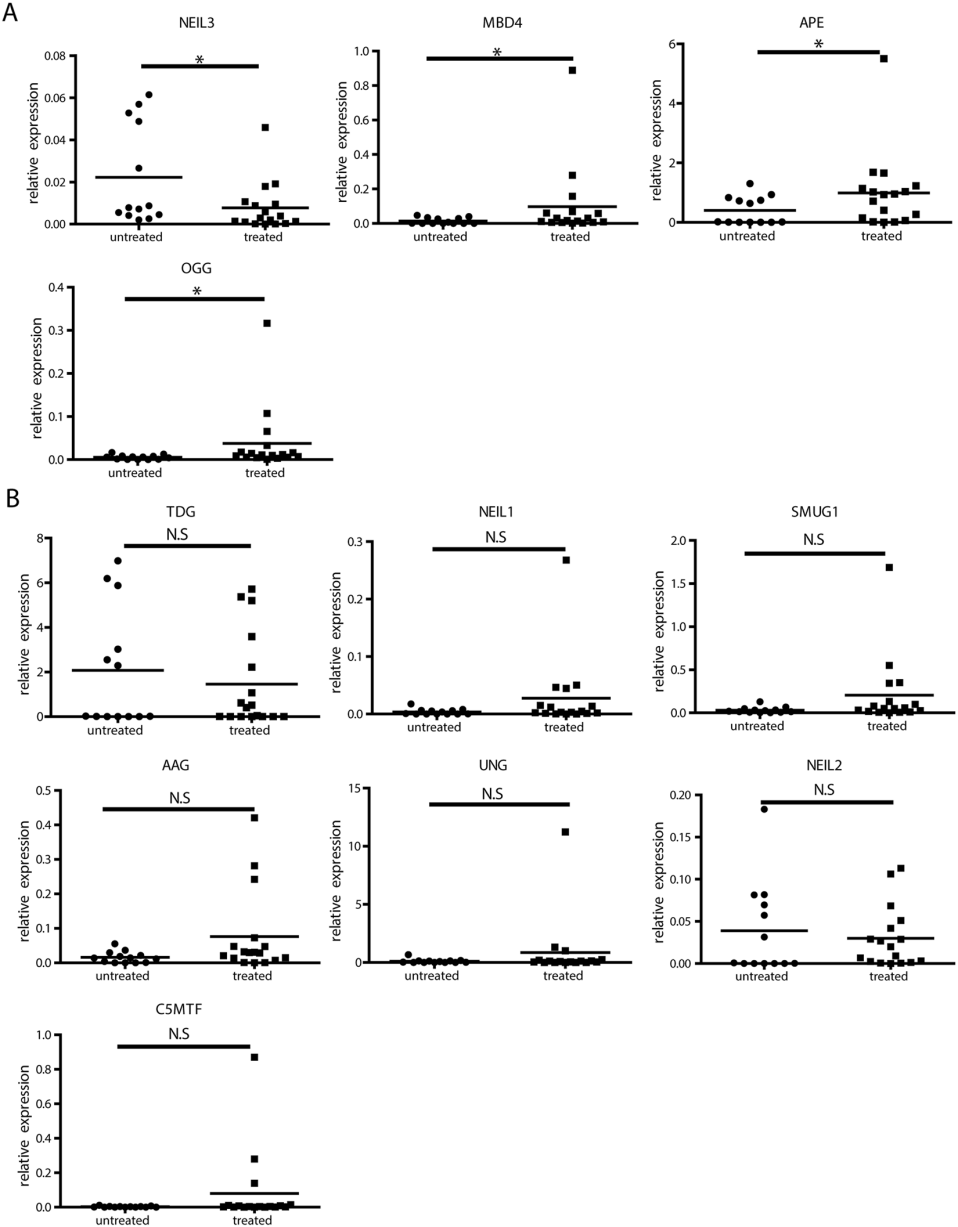
Expression of BER genes in liver biopsies from CHB patients. Total RNA from liver biopsies of naïve or IFN-α-treated CHB patients were extracted. After reverse transcription, (**A**) NEIL3 and TDG mRNA levels and (**B**) NEIL1, NEIL2, MBD4, AAG, OGG, SMUG1, UNG, and APE expression levels were quantified and normalized to *GAPDH* mRNA. Student’s *t* test or Mann Whitney U test test was used to compare the two groups.*P < 0.05; N.S., no significance.

Because deaminase expression on its own will not be sufficient to lead to HBV DNA degradation or loss, we assessed the expression of other genes involved in the DNA BER pathway in liver samples from IFN-α treated and non-treated CHB patients. Interestingly, we found methyl-CpG binding domain 4 DNA glycosylase (MBD4), APEX nuclease (APE) and 8-Oxoguanine DNA Glycosylase (OGG) were induced, while Nei-like DNA glycosylase (NEIL)3 was reduced in liver samples from patients after IFN-α treatment (Fig. 4A). Thymine DNA glycosylase (TDG), NEIL1, NEIL2, N-methylpurine DNA glycosylase (AAG), single-strand-selective mono functional uracil-DNA glycosylase 1 (SMUG1), uracil DNA glycosylase (UNG) and DNA-(cytosine-C5)-methyl transferase (C5MTF) were expressed at comparable levels between naïve- and IFN-α-treated CHB patients (Fig. 4B). As expected, paired liver biopsy samples before and after IFN treatment showed similar pattern as shown in Fig. S3: MBD4, APE and OGG were up-regulated while NEIL3 was down-regulated. However, TDG and NEIL2 were also regulated in this cohort.

Comparing responders to non-responders to IFN-α treatment, NEIL3 and TDG were down-regulated in responders. AAG, SMUG1, OGG and APE mRNA levels showed a tendency to be up-regulated in responders, while NEIL2 and MBD4 mRNA levels showed a tendency to be down-regulated in responders (Fig. S4).

**Figure 5:**
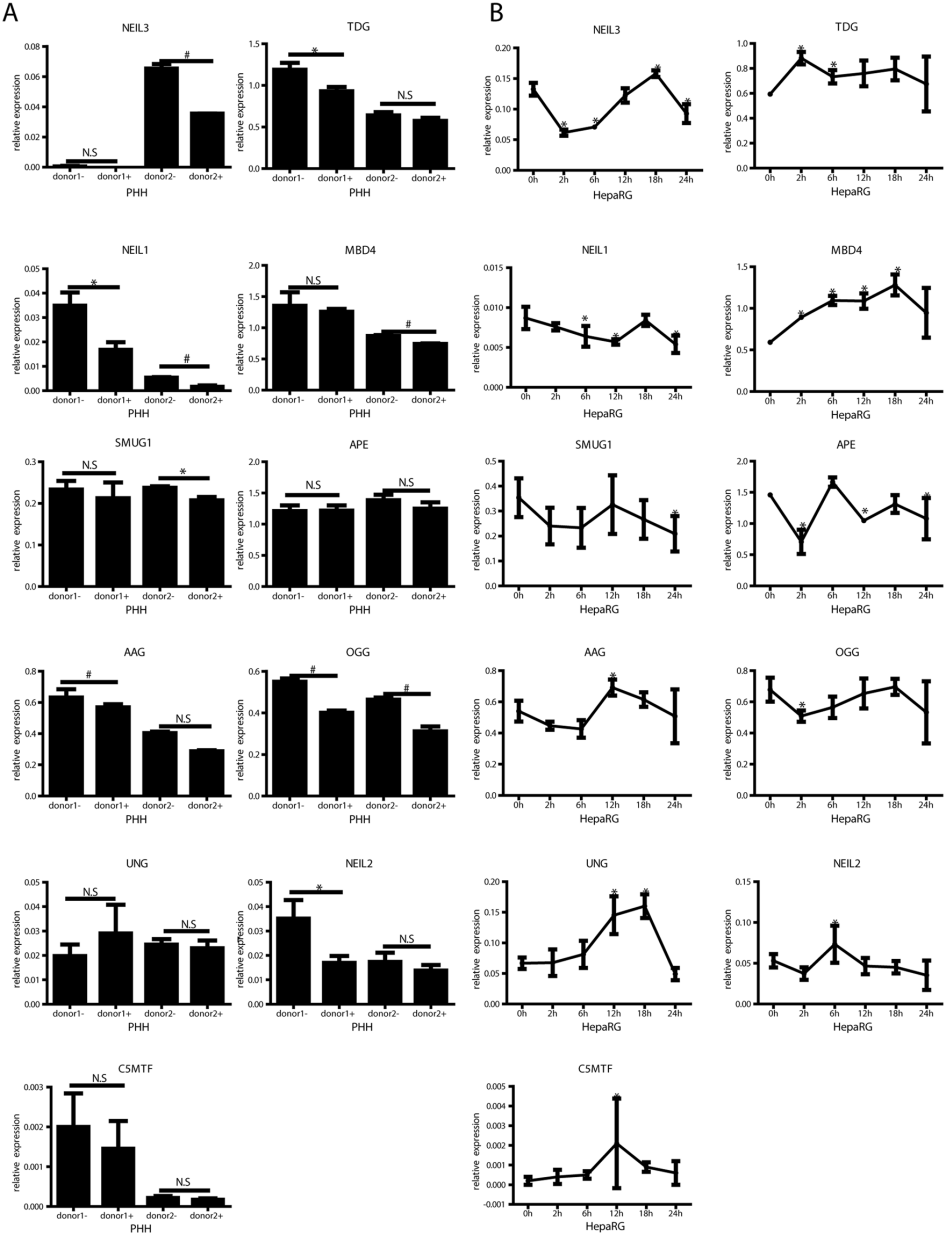
Interferon regulation of BER genes *in cell culture*. PHH (**A**) and differentiated HepaRG cells (**B**) were infected with HBV at MOI 200 virions/cell. After 7 days, cells were treated with 1000 IU/ml IFN-α. Total RNA from PHH and HepaRG cells were extracted after 18 hours (**A**) or at indicated time points (**B**). After reverse transcription, selected mRNAs of enzymes involved in BER were quantified and normalized to *GAPDH* mRNA. ^*^ *P* < 0.05; ^#^ *P* < 0.01; N.S., no significance. Student’s *t* test was used to compare the untreated (donor1/2-) and treated (donor1/2+) PHH. ANOVA was used to compare gene expression at different time points and baseline in HepaRG cells.

To confirm the results of BER enzyme expression from CHB patient samples, PHH and differentiated HepaRG cells were *in vitro* infected with HBV and then treated with IFN-α. As shown in Fig. 5, NEIL3 expression was down-regulated in PHH and HepaRG cells following IFN-α treatment (Fig. 5), while TDG was down-regulated in PHH (Fig. 5A), but up-regulated in HepaRG cells (Fig. 5B). In addition, AAG was down-regulated in PHH but up-regulated in HepaRG cells with IFN-α treatment, while NEIL1 and OGG was down-regulated in both models. UNG and MBD4 were upregulated in HepaRG cells but not in PHH. In summary, besides down-regulation of NEIL3 no distinct regulation of enzymes involved in BER was detected.

### BER enzymes expression correlated with virologic response in PegIFN-α treated CHB patients

**Figure 6:**
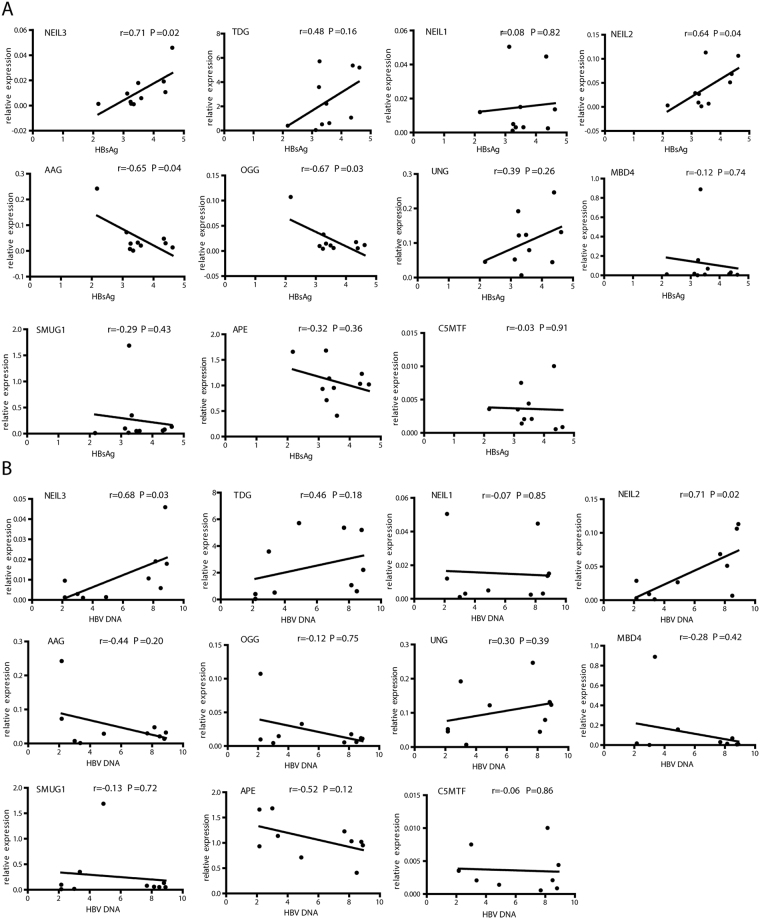
Correlation between BER gene expression and corresponding viral markers. Hepatic expression of BER enzymes at treatment endpoint were correlated with HBsAg (**A**) or HBV DNA (**B**) titers in the serum at treatment endpoint. Correlations between BER gene expression and viral markers were tested using Pearson correlation. Log 10 values of HBsAg or HBV DNA were bloted.

To further investigate whether the expression of enzymes involved in BER correlated with IFN-α treatment response, BER gene expression and HBV markers determined at treatment endpoint were correlated. In patients undergoing IFN-α mono-treatment, hepatic NEIL2 and NEIL3 mRNA levels at treatment endpoints significantly correlated positively to corresponding serum HBsAg levels (Fig. 6A) and HBV viremia (Fig. 6B). AAG and OGG mRNA negatively correlated with HBsAg levels (Fig. 6A) but not with HBV DNA. (Fig. 6B). Interestingly, none of these BER genes showed a significant correlation to HBsAg or HBV DNA decline (Fig. S5). Considering IFN responders showed lower amount of NEIL2 and TDG in their livers at end of treatment (Fig. S4), these findings indicate that *BER* may play a role in controlling HBV and in response to IFN-α treatment in CHB patients, and some of the genes have a potential for use as IFN treatment response indicators. Their roles in controlling HBV infection need to be further studied.

## Discussion

IFN-α has been reported to induce the BER pathway leading to the depletion of the HBV persistent form, the cccDNA, *in vitro* ^10,15^. In this study, we found that APOBEC3 family deaminases were up-regulated in liver biopsies from CHB patients treated with IFN-α compared to a treatment-naïve group. This finding corresponds to previously published data from liver samples of chimpanzees and chronic hepatitis C patients undergoing IFN-α treatment^10^. Importantly, we further found that APOBEC3A and APOBEC3B mRNA expression in responders to IFN therapy was significantly higher than in non-responders. Further more, APOBEC3A expression is negatively correlate with HBV infection markers at treatment endpoint. Thus, higher APOBEC3A levels at treatment endpoints suggests a better response to IFN-α during HBV treatment. Our results, to some extent, were in agreement with a study on HCV:APOBEC3A was up-regulated in early responders but not in non-responders in HCV patients^16^. Although the number of clinical samples in our study is small due to limited availability of liver biopsies in CHB patients under or after therapy, APOBEC3A displayed a trend for positive correlation with the degree of HBsAg and HBV DNA decline. In this scenario, it suggested that APOBEC3A plays an important role in the response to IFN-α.

While detection of deaminated cccDNA from patients’ liver biopsies would be ideal, we unfortunately could not detect cccDNA in the liver biopsies available in our study. This might be due to a variety of reasons. First, to increase specificity, our cccDNA qPCR amplicon is rather long (about 1kB)^17,18^, and thus fragmentation of DNA during tissue fixation or extraction very likely affects the PCR reaction. Secondly, after long-term IFN-α treatment lasting 48 or even 96 weeks, the deaminated cccDNA has very likely already been degraded or repaired by cellular enzymes.

Although cccDNA decreasing could not be proven in this study, HBV DNA and in particular HBsAg decline made a loss of cccDNA very likely. Previous clinical investigations reported by Petersen *et al*. had shown that hepatic cccDNA level positively correlated with corresponding HBsAg and HBV DNA levels^7^. We therefore assessed whether APOBEC3 family gene expression correlated with HBV clinical markers. In addition, we made an effort to explore how viral markers correlated to the expression of enzymes involved in BER. Interestingly, not only nuclear APOBEC3A seemed to be of importance, but also other cytoplasmic APOBEC3 family members were upregulated in IFN-treated patients, and APOBEC3C *and F* mRNA levels correlated with the decline of HBV DNA and HBsAg, respectively. It has been reported that APOBEC3F can impact HBV replication in the cytoplasm and may contribute to the non-cytolytic clearance of HBV^12,19^. In addition, Köck *et al*. reported that transfection of HepG2 cells with a plasmid encoding the APOBEC3C protein resulted in abundant G→A mutations in the majority of newly formed HBV genomes^20^.

APOBEC3 A or B proteins are important to induce HBV cccDNA deamination but are not sufficient to lead to viral clearance^15^. To investigate other effectors induced by IFN-α, we checked ten genes involved in the DNA BER pathway and identified two potential candidates. We found that both NEIL3 and TDG mRNA expression were down-regulated in liver samples from patient under IFN-α treatment. TDG is a member of the mismatch uracil glycosylase branch of the monofunctional uracil DNA glycosylase superfamily. TDG can excise uracil and result in abasic site formation in DNA strands, which may subsequently be processed by the BER machinery^21,22^. NEIL3 has recently been identified as potential binding partner for oxidized cytosine derivatives^23^. It has been reported that a variation trend of TDG and NEIL3 is consistent in cell models^24^, and TDG has a synergistic effect on accumulation of genomic 5-formylcytosine and 5-carboxylcytosine mediated by NEIL3. Our results in clinical samples were in accordance with these findings. We found that NEIL3 and NEIL2 at treatment endpoints was positively correlated with remaining HBsAg and HBV DNA levels. Our results indicated that a part of BER genes, such as NEIL3 and TDG, may represent potential factors related to IFN-α response in HBV treatment.

A previous study showed changes in BER gene mRNA levels in normal liver tissues and tumor cell lines treated with IFN-α^12^. In an effort to confirm our *in vivo* findings in HBV infected cells, differentiated HepaRG cells and PHH were exploited, which are widely used for a better understanding of hepatic disease pathogenesis^25,26^. NEIL3 was significantly down-regulated by IFN-α in both PHH and HepaRG cells, while TDG was down-regulated only in PHH in our study. The different TDG expression pattern in two cells may due to the transformed nature or influence of biliary like cells in HepaRG cultures^27^. Why these two genes were down-regulated and not induced as one may expect in a first instance may be explained by their involvement in viral DNA repair. Once deaminated, nuclear HBV DNA may undergo two fates: either it is repaired or it is degraded. Thus, down-regulation of TDG and NEIL3 maybe consistent with less cccDNA repair and thus more cccDNA degradation. However, more detailed mechanistic studies will be needed to address this point.

Taken together, our study provides new insights into the IFN-α-mediated regulation of BER genes in livers of CHB patients under IFN-α treatment. Response to IFN-α treatment in CHB patients seems to be associated with a distinct differential intra-hepatic BER gene expression profile, where positive responses are associated with higher levels of hepatic APOBEC3A and lower level of NEIL3 and TDG. However, the exact biological mechanisms underlying the influence of BER genes in IFN-α treatment response remain to be determined. Further studies, both *in vivo* and *in cell culture*, may provide a better understanding of the role of BER in antiviral strategies targeting HBV that can be employed to optimize antiviral treatment.

## Methods

### Study subjects, clinical samples and study design

This research was performed according to the ethical guidelines of the 1975 Declaration of Helsinki and received prior approval from the Institutional Review Boards of Tongji Hospital, Tongji Medical College, Huazhong University of Science and Technology. Written informed consents from the patients were obtained prior to inclusion in the study. Sixteen naïve CHB patients were recruited from 2010 through 2011 at the Tongji Hospital affiliated to Huazhong University of Science and Technology in Wuhan, China. Inclusion criteria for naïve CHB patients in the study were HBV DNA level > 10^4^ copies/ml and HBeAg positivity. Six patients who didn’t receive any treatment were used as a control group (Patient #1–6, shown in Supplementary Table 1). Of the IFN-α monotherapy patients (patient #7–16, shown in Supplementary Table 1) receiving treatment with PegIFN-α2a (Pegasys, Roche, Germany) 180 µg once a week for 24 weeks. At 24 weeks, patients with initial response (HBsAg less than1500 IU/ml and HBV DNA decline larger than 1Log10 copies/ml) continued PegIFN-α therapy until week 48 (Patient #11, 12, 13, 14). Remianing patients continued PegIFN-α therapy until week 96 (Patient #7, 8, 9, 10, 15, 16). Blood samples of IFN treated patients were collected at baseline and at the end of treatment. Liver samples of PegIFN-α treated patients were additionally collected at treatment endpoints. Response to IFN-α treatment was defined as HBV DNA level <1000 copies/ml with HBeAg or HBsAg seroconversion or HBsAg decline>1Log_10_IU/ml at treatment endpoint. Patients not meeting these criteria were classified as non-responders.

Seven paired liver biopsy samples were from The Optimising HBeAg Seroconversion in HBeAg-positive CHB patients with combination and Sequential Treatment of PegIFN alfa-2a and ETV (OSST) study^1^. Patients aged 18–65 years who were HBeAg-positive prior to initiation of ETV treatment, had received ETV treatment for ⩾12 months, had been positive for HBsAg for ⩾6 months prior to enrolment and who had serum HBV DNA ⩽1000 copies/ml and serum HBeAg <100 PEIU/ml followed PegIFN-α therapy 180 μg/week for 48 weeks.

Characteristics of the patients are presented in Supplementary Table 1.

### Microarray analysis

GSE17183 and GSE38598 datasets curated by Insilco DB^28^ was used for identification of over-expressed genes after IFN therapy in HCV patients. For 44 samples in under therapy group, 42 were from GSE17183 and the rest 2 (GSM945917, GSM945916) were retrieved from GSE38598 dataset. All 42 control samples were collected from GSE17183. The analysis was performed on GENE-E platform (http://www.broadinstitute.org/cancer/software/GENE-E/), using the SCAN method for data normalization. Fold changes of APOBEC3 family members between under therapy and before therapy groups were compared to determine if they were differentially expressed. For each gene, the significance of expression difference was estimated by a t test (p-value), and it then corrected for multiple hypotheses testing by computing the false discovery rate (FDR) and the family-wise error rate (FWER). The FDR(BH) indicated the FDR value was estimated using the Benjamini and Hochberg procedure^29^.

### Markers characterizing HBV infection

HBsAg, HBeAg and HBV DNA levels were measured at a central laboratory with the ARCHITECT HBsAg assay (Abbott Laboratories, Lake Forest, IL, USA), AxSYM HBe 2.0 assay (Abbott), and either a standard generic HBV DNA assay (ACON Biotech Co. Ltd, Hangzhou, China) or the COBAS TaqMan HBV Test (Roche Molecular Diagnostics, Pleasanton, CA, USA). Anti-HBs and anti-HBe were assessed by ARCHITECT qualitative assays (Abbott).Alanine aminotransferase (ALT) was measured using a Hitachi Model 7600 Series Automatic Analyzer (Hitachi, Tokyo, Japan).

### Cell cultures, HBV infection and IFN-α treatments

Cell culture, HepaRG cell differentiation and HBV infection with concentrated supernatant from HepAD38 cells was performed as described^10^. Primary human hepatocytes (PHH) were isolated from surgical liver specimens after informed consent of patients undergoing metastasis resection and seeded onto plastic dishes coated with collagen type IV^30^. Tissue samples and annotated data were obtained and experimental procedures were performed within the framework of the non-profit foundation Human Tissue & Cell Research (HTCR). PHH and differentiated HepaRG cells were infected with HBV at MOI of 200 DNA-containing, enveloped virus particles/cell. After 7 days, cells were treated with 1000 IU/ml IFN-α (Referon-A, Roche) for indicated time periods.

### qRT-PCR

RNA from liver biopsies was extracted using Trizol reagent (Invitrogen, Carlsbad, CA) and reverse-transcribed into cDNA with QPK201T reverse transcriptase (Toyobo, Tokyo, Japan). RNA from cultured cells was extracted using NucleoSpin® RNA II kit (Macherey Nagel, Düren, Germany) and reverse-transcribed with the Superscript III Reverse Transcription kit (Invitrogen, Carlsbad, USA) according to manufacturer’s instructions. Expression levels were quantified by qRT-PCR performed on a 7500 HT qRT-PCR system (Applied Biosystems, Life Technologies, Darmstadt, Germany) or on a LightCycler™ system (Roche Diagnostics, Basel, Switzerland) and analyzed by the second derivative maximum method that includes both normalization to the reference genes and primer efficiency.

RNA from liver biopsies and cultured cells was extracted, reverse transcribed and analyzed by qPCR (details see Supplementary Methods). Relative mRNA levels of all target genes were normalized to GAPDH house-keeping genes. The primers of target genes are listed in Supplementary Table 2.

### Statistical analysis

The results were analyzed by the Student’s t test, Mann Whitney U test or ANOVA where appropriate. Distribution of the samples was evaluated by Kolmogorv- Smirnov test. The correlation of target gene and HBV clinical markers was processed by Pearson correlation. All statistical analyses were carried out with SPSS v.11 (SPSS, Chicago USA). *P* value of less than 0.05 was considered statistically significant.

## Electronic supplementary material

Supplementary Information
