# Supplementary material for: IFN-α-mediated Base Excision Repair Pathway Correlates with Antiviral Response Against Hepatitis B Virus Infection
Source: Sci Rep. 2017 Oct 5;7:12715. doi: 10.1038/s41598-017-13082-z (PMC5629255; doi:10.1038/s41598-017-13082-z)
Supplement: Supplementary file 1 — Supplementary Information [file 41598_2017_13082_MOESM1_ESM.docx]

**IFN-α-mediated Base Excision Repair Pathway Correlates with Antiviral Response Against Hepatitis B Virus Infection**

Yong Li^1^*, Yuchen Xia^2,3^*, Meifang Han^1^, Guang Chen^1^,Dake Zhang^4^, Wolfgang E. Thasler^5^, Ulrike Protzer^2,6&^, Qin Ning^1&^

^1^Department and Institute of Infectious Diseases, Tongji Hospital, Tongji Medical College, Huazhong University of Science and Technology, Wuhan 430030, China

^2^Institute of Virology, Technical University of Munich / Helmholtz Zentrum München, 81675 Munich, Germany

^3^Liver Diseases Branch, National Institute of Diabetes and Digestive and Kidney Diseases (NIDDK), NIH, 20892 Bethesda, Maryland, USA

^4^Key Laboratory of Genomic and Precision Medicine, Beijing Institute of Genomics, Chinese Academy of Sciences, Beijing 100101, China

^5^Department of General, Visceral, Transplantation, Vascular and Thoracic Surgery, Grosshadern Hospital, Ludwig Maximilians University, 81377 Munich, Germany

^6^German Center for Infection research (DZIF)

* These authors contributed equally to this work.

^&^ These authors contributed equally to this work.

Corresponding author:

Prof. Qing Ning, Department and Institute of Infectious Diseases, Tongji Hospital, Tongji Medical College, Huazhong University of Science and Technology, Wuhan 430030, China Tel: Email:[qning@vip.sina.com](mailto:qning@vip.sina.com).

**
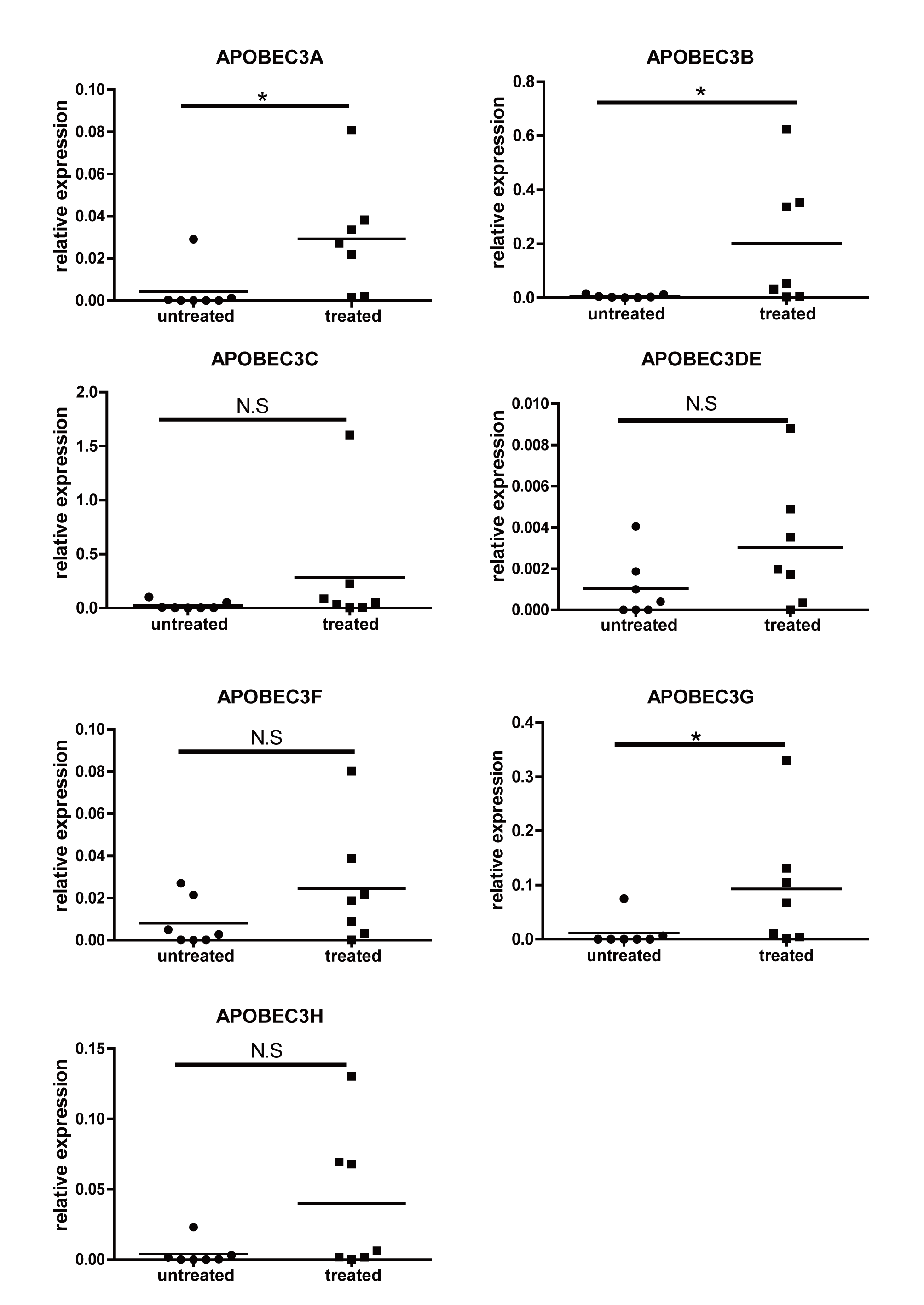
**

**FigS1.** **Comparison of APOBEC3 gene expression before and after IFN therapy.**

Total RNA from liver biopsies of OSST patients before and after IFN therapy (paired samples) were extracted. After reverse transcription, mRNA expression levels of APOBEC3 genes were quantified and normalized to GAPDH mRNA. N.S, no significance.

**
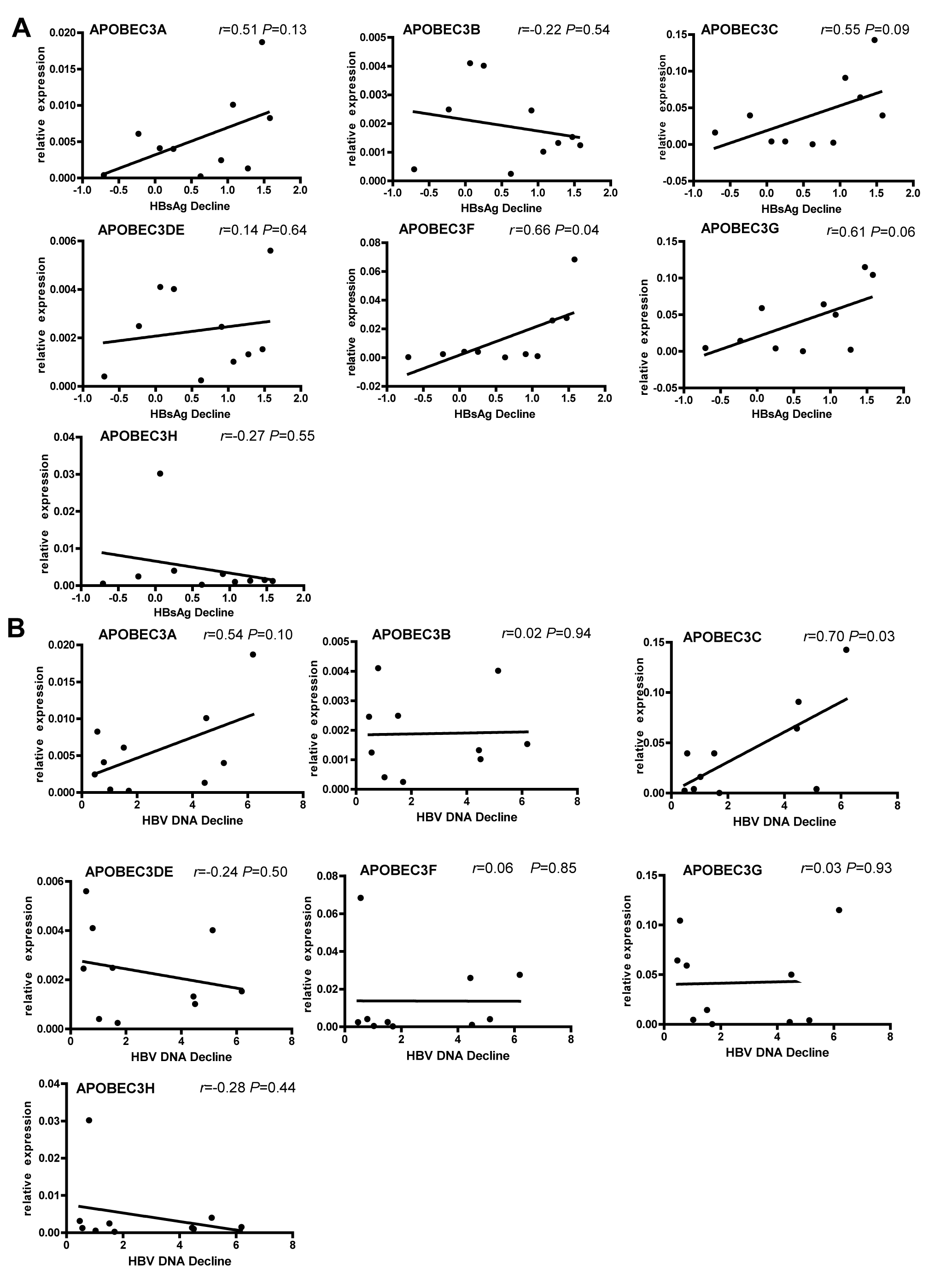
**

**FigS2. Correlation between *APOBEC3* gene expression and degree of HBV viral marker decline from start of therapy to end of therapy.**

Total RNA from liver biopsies of CHB patients treated with IFN-α were extracted at treatment endpoints at week 48 or 96, respectively. After reverse transcription, correlation between *APOBEC3* mRNA level at treatment endpoints and corresponding (Log10) decline of HBsAg (A) and HBV DNA (B) were studied.


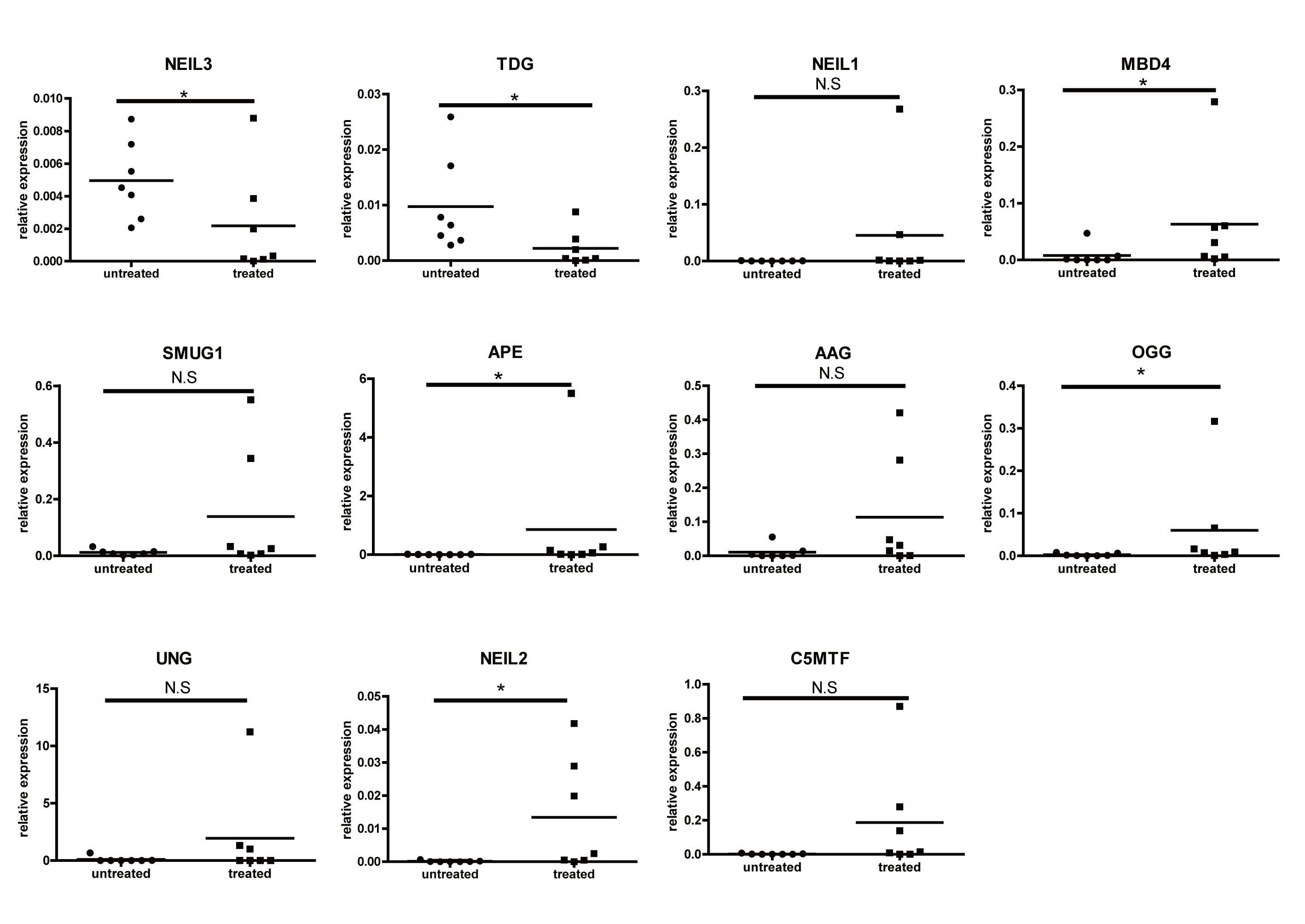


**FigS3.** **Comparison of BER gene expression before and after IFN therapy.**

Total RNA from liver biopsies of OSST patients before and after IFN therapy (paired samples) were extracted. After reverse transcription, mRNA expression levels of selected genes NEIL1, NEIL2, NEIL3, TDG, AAG, OGG, UNG, MBD4, SMUG1,APE and C5MTF were quantified and normalized to GAPDH mRNA. N.S., no significance.

**
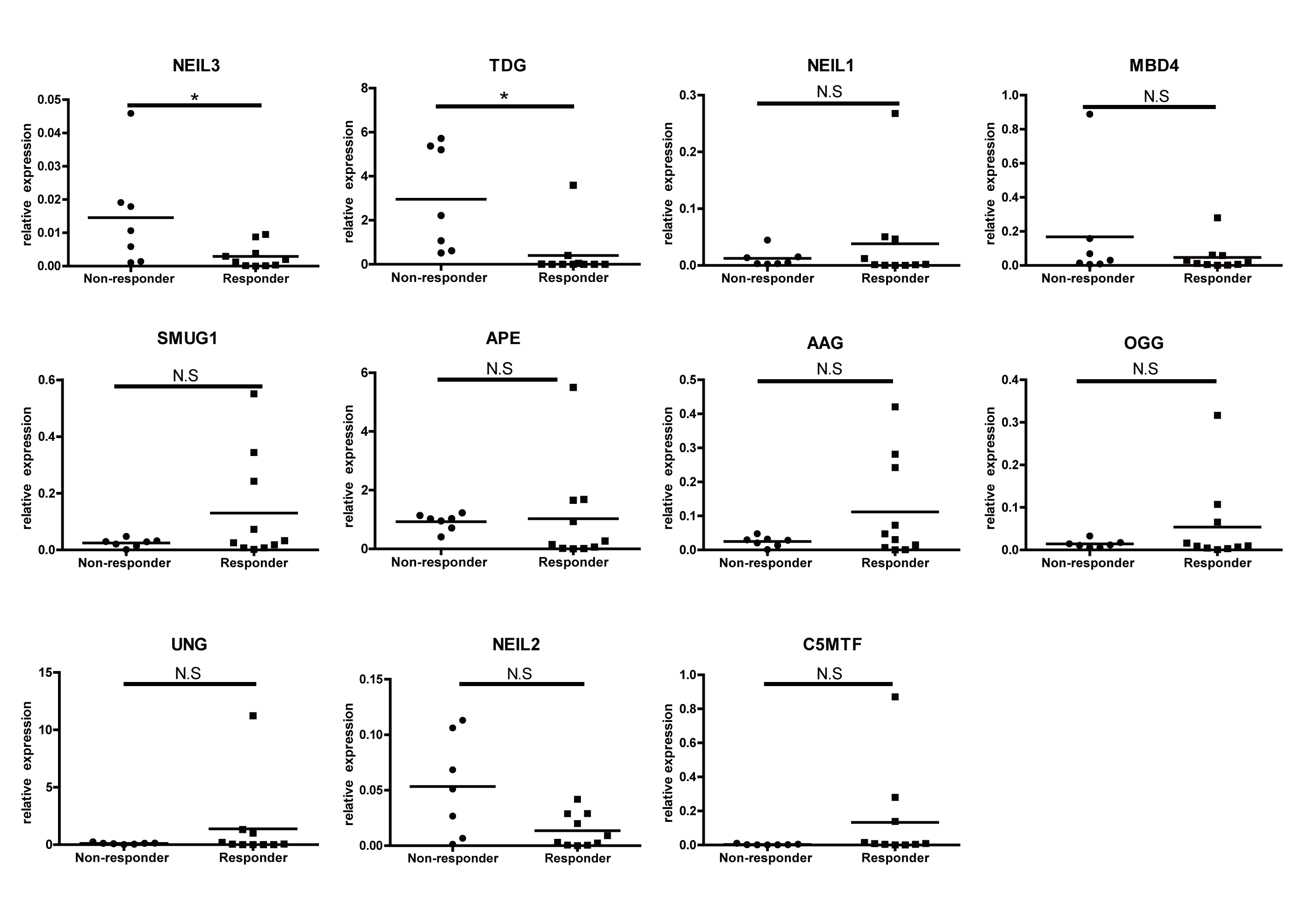
**

**FigS4.** **Comparison of BER gene expression between responders and non-responders.**

Total RNA from liver biopsies of responders or non-responders to IFN therapy at treatment endpoint were extracted. After reverse transcription, mRNA expression levels of selected genes NEIL1, NEIL2, NEIL3, TDG, AAG, OGG, UNG, MBD4, SMUG1, APE and C5MTF were quantified and normalized to GAPDH mRNA. N.S, no significance.


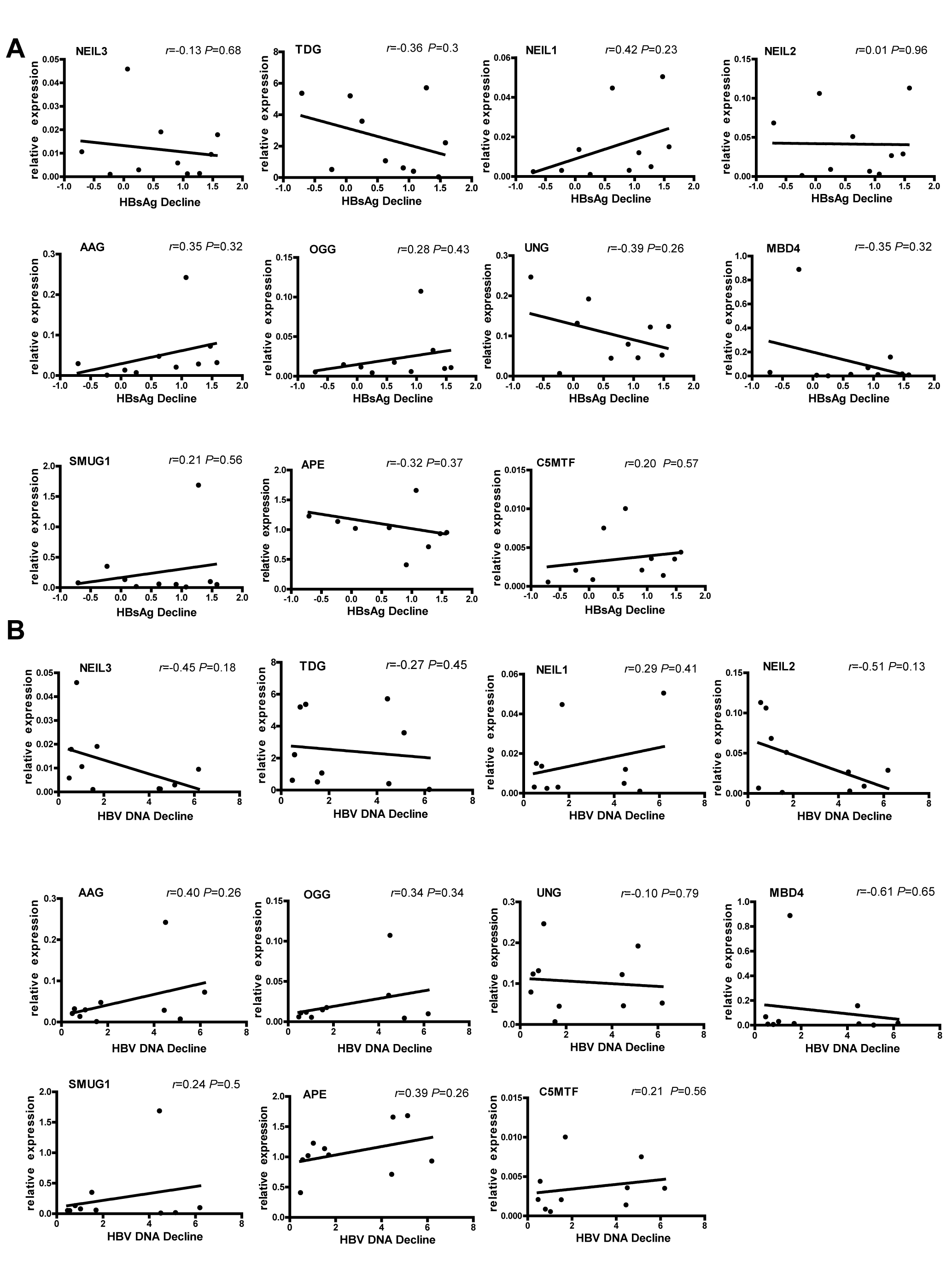


**FigS5. Correlation between BER gene expression and degree of HBV viral marker decline from start of therapy to end of therapy.**

Total RNA from liver biopsies of CHB patients treated with IFN-α were extracted at treatment endpoints. After reverse transcription, correlation between BER gene mRNA level at treatment endpoints and corresponding HBsAg/HBV DNA(Log10) decline were studied.

**Supplementary Table 1: Baseline characteristics of patients included in the study**

Treatment naïve patients:

| Patient | Gender | Age | HBV DNA  (Log_10_ copies/ml) | HBsAg  (IU/ml) | HBeAg  (PEIU/ml) | ALT  (IU/ml) | Treatment  duration |
| --- | --- | --- | --- | --- | --- | --- | --- |
| 1 | F | 52 | 8.33 | 1233.68 | 353.42 | 42 | 0 |
| 2 | F | 31 | 8.72 | 16495.55 | 1278.99 | 104 | 0 |
| 3 | M | 23 | 8.13 | 91323.66 | 7033.30 | 63 | 0 |
| 4 | M | 23 | 6.52 | 3421.23 | 6837.12 | 65 | 0 |
| 5 | M | 16 | 7.90 | 3521.32 | 6521.32 | 52 | 0 |
| 6 | M | 42 | 5.41 | 2892.11 | 6345.26 | 38 | 0 |

IFN monotherapy patients:

| Patient | Gender | Age | HBV DNA  (Log_10_ copies/ml) | HBsAg  (IU/ml) | HBeAg  (PEIU/ml) | ALT  (IU/ml) | Treatment  duration |
| --- | --- | --- | --- | --- | --- | --- | --- |
| 7 | M | 29 | 9.59 | 48222.02 | 1085.97 | 76 | 96w |
| 8 | M | 31 | 8.72 | 4829.68 | 101.76 | 105 | 96w |
| 9 | M | 31 | 8.96 | 31612.60 | 2270.30 | 152 | 96w |
| 10 | M | 26 | 9.85 | 91440.48 | 7037.30 | 67 | 96w |
| 11 | M | 23 | 8.35 | 39633.77 | 102.45 | 82 | 48w |
| 12 | M | 23 | 6.66 | 1752.98 | 0.24 | 54 | 48w |
| 13 | M | 28 | 9.45 | 118277.16 | 2831.01 | 80 | 48w |
| 14 | M | 33 | 4.88 | 1287.65 | 0.22 | 34 | 48w |
| 15 | F | 27 | 9.33 | 34298.81 | 473.07 | 146 | 96w |
| 16 | M | 23 | 8.14 | 3101.09 | 5.42 | 87 | 96w |

OSST patients (paired samples):

| Patient | Gender | Age | HBV DNA  (Log_10_ copies/ml) | HBsAg  (IU/ml) | HBeAg  (PEIU/ml) | ALT  (IU/ml) | Treatment  duration |
| --- | --- | --- | --- | --- | --- | --- | --- |
| 17 | F | 27 | <1x10^3^ | \| 140.12 \| \| --- \| | 0.1 | 32 | 48w |
| 18 | M | 25 | <1x10^3^ | 30.81 | 0.1 | 11 | 48w |
| 19 | M | 20 | <1x10^3^ | 512.48 | 0.1 | 8 | 48w |
| 20 | M | 42 | <1x10^3^ | 2584.11 | 0.7 | 16 | 48w |
| 21 | F | 21 | <1x10^3^ | 837.05 | 1.6 | 10 | 48w |
| 22 | M | 38 | <1x10^3^ | 936.83 | 0.1 | 15 | 48w |
| 23 | M | 21 | <1x10^3^ | 1945.1 | 0.4 | 23 | 48w |

**Supplementary Table 2: qRT-PCR primers for target genes**

| Genes | Forward | Reverse |
| --- | --- | --- |

SMUG1 ctgcagtgcctgtcatgtg gcaggctcatggatggac
TDG gaaccttgtggcttctcttca gtcatccactgcccattagg
MBD4 ggcaacgactcttaccgaat cccaaagccagtcatgatattt
OGG1 ctgcatcctgcctggagt gcctggggcttgtctagg
AAG tttacggcatgtacttctgcat atggtctccagaccttccag
NEIL1 gaccaagctgcagaatccag tctctgacccgtagcctttg
NEIL2 ccaggacacccaggtgag gggaagagctccaagacaact

NEIL3 cctccacggttgtggtct aagacattctggctggaatca

UNG2 tcgcttcctggcggg gccagaagacgctctactcc

APE ttggtctctcttgaaggcacagt gcttcgagcctggattaaga

C5MTF ggaaattagaatcaaggaaatacga aatttgtcttgaggcgcttg

GAPDH cggatttggtcgtattggg ctcgctcctggaagatgg

**Supplementary Table 3. Differentially expression of APOBEC3A-G in tissues from under and before therapy patients.**

| *ID* | *FoldChange* | *p-value* | *FDR(BH)* | *Bonferroni* | *FWER* |
| --- | --- | --- | --- | --- | --- |
| APOBEC3A | 14.046733 | 0.001996008 | 0.00249505 | 0.029940119 | 0 |
| APOBEC3B | 77.38516 | 0.001996008 | 0.00249505 | 0.029940119 | 0 |
| APOBEC3C | 2.8027709 | 0.001996008 | 0.00249505 | 0.029940119 | 0 |
| APOBEC3DE | 1.6969877 | 0.16766468 | 0.16766468 | 1 | 0.802 |
| APOBEC3F | -11.318149 | 0.06986022 | 0.07485024 | 1 | 0.443 |
| APOBEC3G | -1.3463254 | 0.015968084 | 0.018424712 | 0.23952127 | 0.078 |

FDR(BH), false discovery rate estimated using the Benjamini and Hochberg procedure; FWER, family-wise error rate; *Bonferroni*, p value after Bonferroni correction.
